# Bulk and Single-Cell Transcriptomics Reveal That SCO2 Drives Psoriasis via Activating CCR7^+^ Dendritic Cell

**DOI:** 10.3390/ijms27031397

**Published:** 2026-01-30

**Authors:** Donger Chen, Jing Yang, Guoliang Zhou, Xiaoqing Xu, Yuekang Zhang, Yanting Duan, Bin Liu, Zhuo Zhu, Fusheng Zhou

**Affiliations:** 1 Department of Dermatology, The First Affiliated Hospital, Anhui Medical University, Hefei 230032, China; 2345011374@stu.ahmu.edu.cn (D.C.); 2445011410@stu.ahmu.edu.cn (J.Y.); 2445011406@stu.ahmu.edu.cn (G.Z.); 2446010151@stu.ahmu.edu.cn (X.X.); 2445011405@stu.ahmu.edu.cn (Y.Z.); 2445011409@stu.ahmu.edu.cn (Y.D.); 2445011640@stu.ahmu.edu.cn (B.L.); 2 Institute of Dermatology, Anhui Medical University, Hefei 230032, China; 3 Key Laboratory of Dermatology (Anhui Medical University), Ministry of Education, Hefei 230032, China; 4 Inflammation and Immune Mediated Diseases Laboratory of Anhui Province, Hefei 230032, China

**Keywords:** psoriasis, SCO2, Macrophage Migration Inhibitory Factor (MIF), CCR7^+^ dendritic cells, lactate, machine learning

## Abstract

Metabolic reprogramming is a hallmark of psoriasis, yet the contribution of lactate metabolism to keratinocyte-mediated immune dysregulation remains undefined. Through integrated bulk and single-cell RNA sequencing, validated by immunofluorescence and metabolic assays, we identified the mitochondrial protein SCO2 as a key pathogenic hub gene upregulated in psoriatic lesions. Functionally, SCO2 overexpression promoted keratinocyte migration and triggered a metabolic shift characterized by mitochondrial pyruvate accumulation and intracellular lactate retention. Single-cell analysis further revealed that SCO2-high keratinocytes establish pathogenic crosstalk with CCR7^+^ dendritic cells via MIF-(CD74 + CD44) interactions, wherein these CCR7^+^ dendritic cells serve as the primary source of IL-23 and co-stimulatory signals (CD80/CD86) to drive robust T cell priming. Our findings highlight SCO2 as a pivotal immunometabolic switch linking keratinocyte metabolism to adaptive immunity. Targeting SCO2 offers a novel strategy to disrupt the keratinocyte-driven recruitment of CCR7^+^ DCs, thereby attenuating the IL-23-mediated inflammatory cascade. Furthermore, SCO2 may serve as a potential biomarker for metabolic dysregulation in psoriatic lesions.

## 1. Introduction

Psoriasis is a common, chronic, immune-mediated skin disorder characterized by keratinocyte hyperproliferation, aberrant differentiation, and prominent immune cell infiltration [1,2]. Pathogenesis entails reciprocal keratinocyte-adaptive immune crosstalk, wherein the IL-23/Th17 axis orchestrates the inflammatory response [3,4]. Despite clinical success of cytokine-targeted biologics, the upstream molecular events in keratinocytes that initiate or sustain this pathogenic immune response remain poorly defined [5].

Growing evidence links psoriasis with metabolic disorders, establishing metabolic reprogramming as a disease hallmark and key research focus [6,7]. Prior studies have revealed that energy competition occurs among epithelial cells in psoriasis, significantly complicating the metabolic landscape of the epidermis [8]. Beyond a metabolic byproduct, lactate exerts important biological effects: it directly suppresses cytotoxic CD8^+^ T cells by lowering intracellular pH to inhibit their proliferation and function, while also acting as a bioactive signaling molecule that orchestrates immune responses [9]. However, the mitochondrial checkpoints governing metabolic plasticity and their translation into keratinocyte-mediated intercellular immune crosstalk remain poorly defined.

Synthesis of cytochrome c oxidase 2 (SCO2) is a copper metallochaperone essential for the assembly of the mitochondrial cytochrome c oxidase (COX) complex [10,11]. As one of eight COX metallation proteins, SCO2 delivers copper to the CuA site of Complex IV and serves as a redox sensor during cellular stress [12]. Mutations in the SCO2 gene are therefore linked to severe mitochondrial disorders, including fatal infantile cardioencephalomyopathy [13]. SCO2 additionally operates as a key metabolic checkpoint that modulates the balance between mitochondrial respiration and glycolysis. RelA induces SCO2 expression, promoting oxidative phosphorylation (OXPHOS) and driving M2 polarization of tumor-associated macrophages (TAMs) [14]. SCO2, a direct p53 transcriptional target, modulates cellular responses to metabolic stress and reactive oxygen species (ROS) [15]. In p53-deficient cells, reduced SCO2 expression facilitates a metabolic shift from OXPHOS to glycolysis, contributing to the Warburg effect in cancer [15,16]. SCO2-mediated ROS regulation is cell context-dependent: silencing reduces ROS in MCF-7 and KB cells [17], whereas deficiency in HCT116 cells impairs mitochondrial respiration and redox homeostasis, paradoxically increasing ROS generation and oxidative DNA damage [18]. Despite its pivotal metabolic role, the expression pattern of SCO2 and its potential function in linking metabolic rewiring to immune cell recruitment in psoriasis remain unexplored.

In this study, we aimed to bridge the gap between metabolic reprogramming in keratinocytes and immune cell recruitment in psoriasis. By integrating single-cell RNA sequencing with experimental validation, we identified SCO2 as a pivotal lactate-related regulator significantly upregulated in psoriatic lesions. Functionally, we demonstrate that SCO2 overexpression drives a metabolic shift in lactate and pyruvate distribution, thereby promoting keratinocyte migration. Mechanistically, we uncover a novel pathway wherein SCO2-high keratinocytes secrete Macrophage Migration Inhibitory Factor (MIF) to recruit and activate pathogenic CCR7^+^ dendritic cells (DCs), which may facilitate T cell activation via IL-23 secretion and co-stimulation. To our knowledge, this study provides the first evidence of the SCO2-MIF-CCR7^+^ DC crosstalk, establishing SCO2 as a critical immunometabolic switch that links mitochondrial dysfunction to the adaptive immune response in psoriasis.

## 2. Results

### 2.1. Differential Gene Expression and Lactate-Related Pathway Enrichment Analyses in Psoriasis

**Figure 1:**
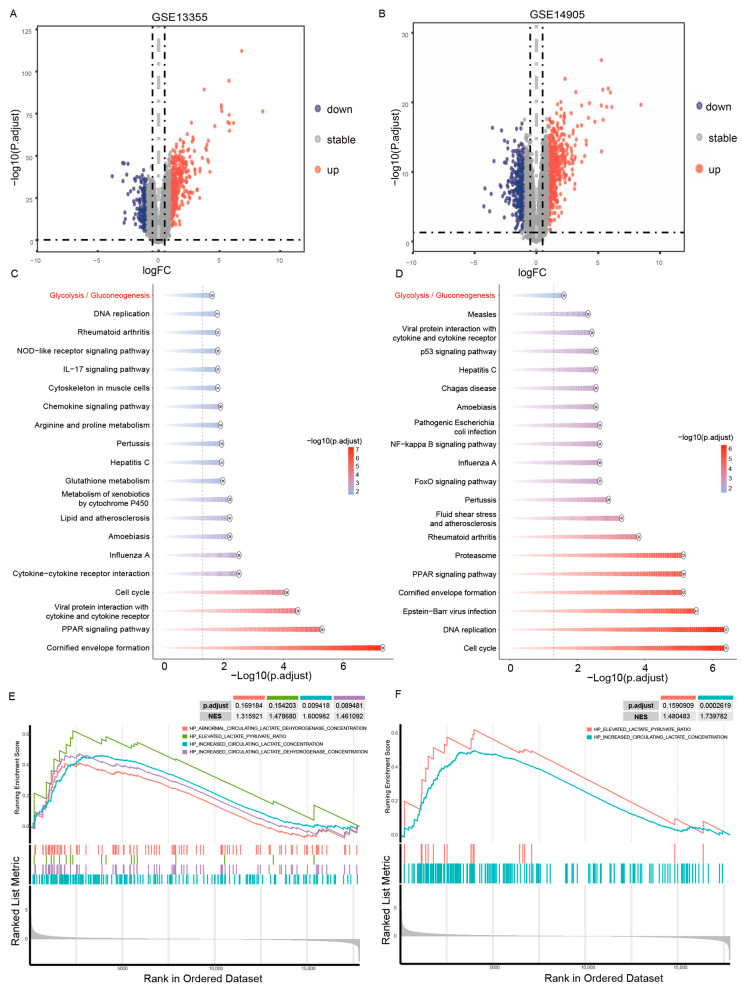
Transcriptomic profiling reveals dysregulated lactate metabolism in psoriasis. (**A**,**B**) Volcano plots showing differentially expressed genes (DEGs) between psoriatic lesional skin (PS) and normal skin (NS) in GSE13355 (**A**) and GSE14905 (**B**). The horizontal black dashed line indicates the statistical significance threshold (P.adj < 0.05). The two vertical black dashed lines represent the biological fold-change thresh-olds (∣FoldChange∣ > 0.5). The central grey vertical dashed line indicates FoldChange = 0. Red, blue, and grey dots represent significantly upregulated, downregulated, and non-significantly expressed genes, respectively. (**C**,**D**) KEGG pathway enrichment analysis of DEGs in GSE13355 (**C**) and GSE14905 (**D**). The color scale represents the adjusted *p*-value (*p*.adjust), and the number inside each circle denotes the number of enriched genes. The “Glycolysis/Gluconeogenesis” pathway is highlighted in red. The vertical dashed line represents the statistical significance threshold at p.adjust = 0.05. (**E**,**F**) Gene Set Enrichment Analysis (GSEA) demonstrating the significant enrichment of lactate-related gene signatures in GSE13355 (**E**) and GSE14905 (**F**). The Normalized Enrichment Score (NES) and adjusted *p*-values (FDR) are indicated. Pathways with an FDR < 0.25 were considered significantly enriched.

Two independent microarray datasets (GSE13355 and GSE14905) were analyzed to characterize psoriasis transcriptomic alterations. Principal component analysis demonstrated clear segregation between PS and NS samples in both datasets, confirming disease-specific expression profiles (Supplementary Figure S1A,B). Differential expression analysis identified 1148 upregulated and 1038 downregulated genes in GSE13355, and 1641 upregulated and 1407 downregulated genes in GSE14905 (Figure 1A,B). To evaluate the reproducibility of these transcriptomic signatures, we performed an intersection analysis of the DEGs from both cohorts. As shown in Supplementary Figure S1C, a substantial overlap of 1592 genes was identified between the two datasets. A hypergeometric test confirmed that this overlap was highly statistically significant (*p* < 2.2 × 10^−16^), underscoring the robustness and reliability of the identified disease-associated gene signatures. To elucidate the biological functions of the identified DEGs, KEGG pathway enrichment analysis was performed. Notably, the “Glycolysis/Gluconeogenesis” pathway emerged as one of the most significantly enriched pathways for upregulated genes in both the GSE13355 (Figure 1C) and GSE14905 (Figure 1D) datasets, indicative of a metabolic shift toward glycolysis in psoriatic skin (PS). Additionally, other pathways implicated in psoriasis pathogenesis, such as the IL-17 signaling pathway, were also significantly enriched among the upregulated genes in these datasets. In light of the emerging roles of lactylation and lactate metabolism in inflammatory diseases, we conducted GSEA to investigate pathways related to lactylation and lactate metabolism. In the GSE13355 dataset, we observed positive enrichment for gene sets associated with abnormal circulating lactate dehydrogenase concentration, an elevated lactate-pyruvate ratio, and increased circulating lactate and lactate dehydrogenase concentrations (Figure 1E). This pattern was similarly observed in the GSE14905 dataset, which showed significant enrichment for an elevated lactate-pyruvate ratio and increased circulating lactate concentration (Figure 1F). The positive enrichment scores for these gene sets indicate a consistent activation of lactate-associated metabolism in psoriasis. Collectively, these results demonstrate a consistent upregulation of lactate-related genes and metabolic pathways in psoriatic lesions across independent cohorts, suggesting that metabolic reprogramming centered on lactate production and accumulation plays a pivotal role in psoriasis pathogenesis.

### 2.2. Identification of Lactate-Related Genes Through WGCNA in Psoriasis

**Figure 2:**
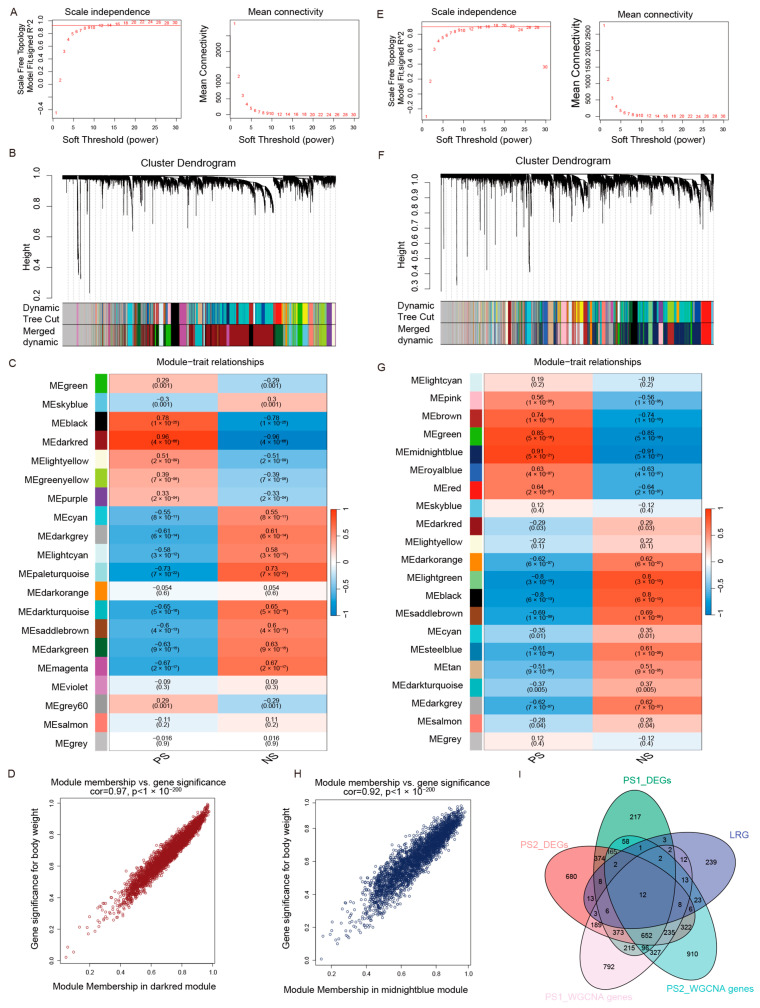
Identification of lactate-related hub genes. (**A**) Analysis of network topology for soft-thresholding powers in GSE13355; the horizontal red line represents the scale-free fit index threshold (R2=0.90). (**B**) Gene clustering dendrogram based on topological overlap for GSE13355, showing the identified co-expression modules. (**C**) Heatmap of module–trait relationships in GSE13355; the darkred module exhibited the strongest positive correlation with psoriasis. (**D**) Scatter plot of Module Membership (MM) vs. Gene Significance (GS) for the darkred module in GSE13355. (**E**) Analysis of network topology for soft-thresholding powers in GSE14905; the horizontal red line similarly indicates the scale-free fit index threshold (R2=0.90). (**F**) Gene clustering dendrogram based on topological overlap for GSE14905, showing the identified co-expression modules. (**G**) Heatmap of module–trait relationships in GSE14905; the midnightblue module exhibited the strongest positive correlation with psoriasis. (**H**) Scatter plot of MM vs. GS for the midnightblue module in GSE14905. (**I**) Venn diagram identifying 12 common candidate genes by intersecting DEGs and key WGCNA module genes from both datasets with lactate-related genes (LRGs).

To identify key gene modules associated with psoriasis, we performed WGCNA on the GSE13355 and GSE14905 datasets. For GSE13355, a soft-thresholding power of β = 12 was chosen to construct a scale-free network topology (Figure 2A), yielding 20 distinct modules (Figure 2B). The “darkred” module showed the strongest positive correlation with psoriasis (r = 0.96, *p* = 4 × 10^−66^) (Figure 2C). The high correlation between gene significance for psoriasis and module membership within this module (cor = 0.97, *p* < 1 × 10^−200^) confirmed its relevance to disease pathophysiology (Figure 2D).

For the GSE14905 dataset, a soft-thresholding power of β = 14 was applied (Figure 2E), generating 21 modules (Figure 2F). The “midnightblue” module exhibited the highest positive correlation with psoriasis (r = 0.85, *p* = 5 × 10^−21^) (Figure 2G). The strong correlation between gene significance and module membership for this module (cor = 0.92, *p* < 1 × 10^−200^) further validated the significance of its highly co-expressed genes for the psoriatic phenotype (Figure 2H).

To identify key hub genes, we intersected five gene sets: differentially expressed genes from GSE13355 (PS1_deg) and GSE14905 (PS2_deg), genes from the most significant psoriasis-associated modules in each dataset (PS1_WGCNA genes and PS2_WGCNA genes, corresponding to the darkred and midnightblue modules, respectively), and lactylation and lactate-related genes (LRG). This intersection yielded 12 genes common to all five groups (Figure 2I).

### 2.3. Machine Learning Algorithms Identify a Core Lactate-Related Gene

**Figure 3:**
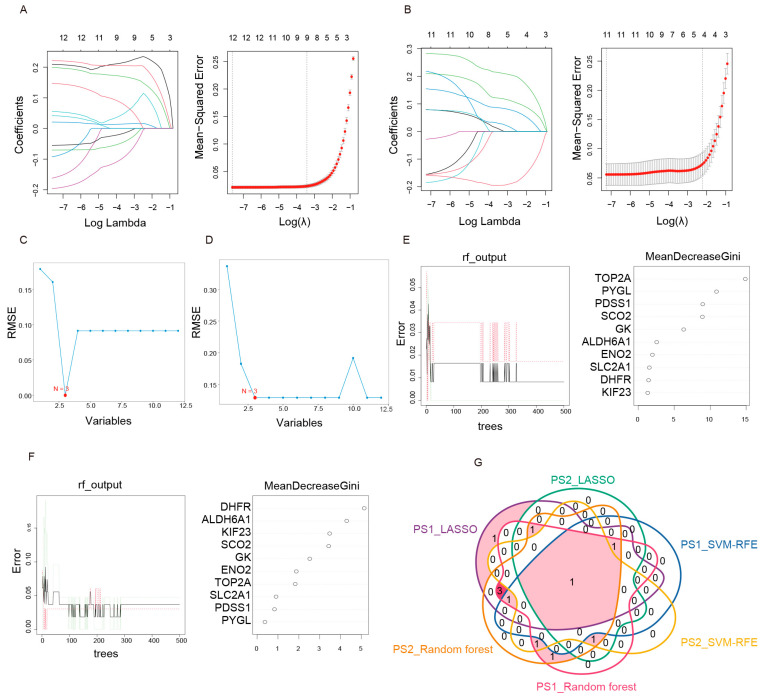
Screening of lactate-related hub genes using multiple machine learning algorithms. (**A**, **B**) LASSO regression analysis in GSE13355 (**A**) and GSE14905 (**B**), including coefficient profiles and cross-validation plots. In the coefficient profiles, each colored line represents the coefficient path of an individual gene as the L1 penalty parameter (λ) varies. In the partial likelihood deviance plots, the vertical dotted lines indicate the optimal λ values selected by cross-validation. (**C**,**D**) SVM-RFE algorithm identifying optimal feature subsets in GSE13355 (**C**) and GSE14905 (**D**) The red dot indicates the number of features with the minimum RMSE. (**E**,**F**) Random Forest analysis displaying error rates and gene importance ranking (MeanDecreaseGini) in GSE13355 (**E**) and GSE14905 (**F**), The error curves represent the relationship between the number of trees and the classification error. The dot plot on the right displays the top 10 genes ranked by MeanDecreaseGini. (**G**) Venn diagram showing the intersection of candidate genes from the three algorithms across both datasets, identifying SCO2 as the unique core hub gene.

We further screened the 12 lactate-related hub genes using three distinct machine learning algorithms applied to both the GSE13355 and GSE14905 datasets. LASSO regression analysis selected eight potential diagnostic markers in the GSE13355 dataset (Figure 3A) and four in the GSE14905 dataset (Figure 3B). Subsequently, the SVM-RFE algorithm identified three key genes in GSE13355 (Figure 3C) and three in GSE14905 (Figure 3D). The RF algorithm, which ranks genes by importance, yielded the top 10 genes for GSE13355 (Figure 3E) and GSE14905 (Figure 3F). We then intersected the results from all six analyses using a Venn diagram to determine the most reliable and central gene (Figure 3G). This intersection revealed that only SCO2 was consistently identified by all three methods across both datasets. Consequently, SCO2 emerges as the core gene for psoriasis among the lactate-related hub genes.

### 2.4. SCO2 Promotes Keratinocyte Migration, Apoptosis, and Intracellular Lactate Accumulation

**Figure 4:**
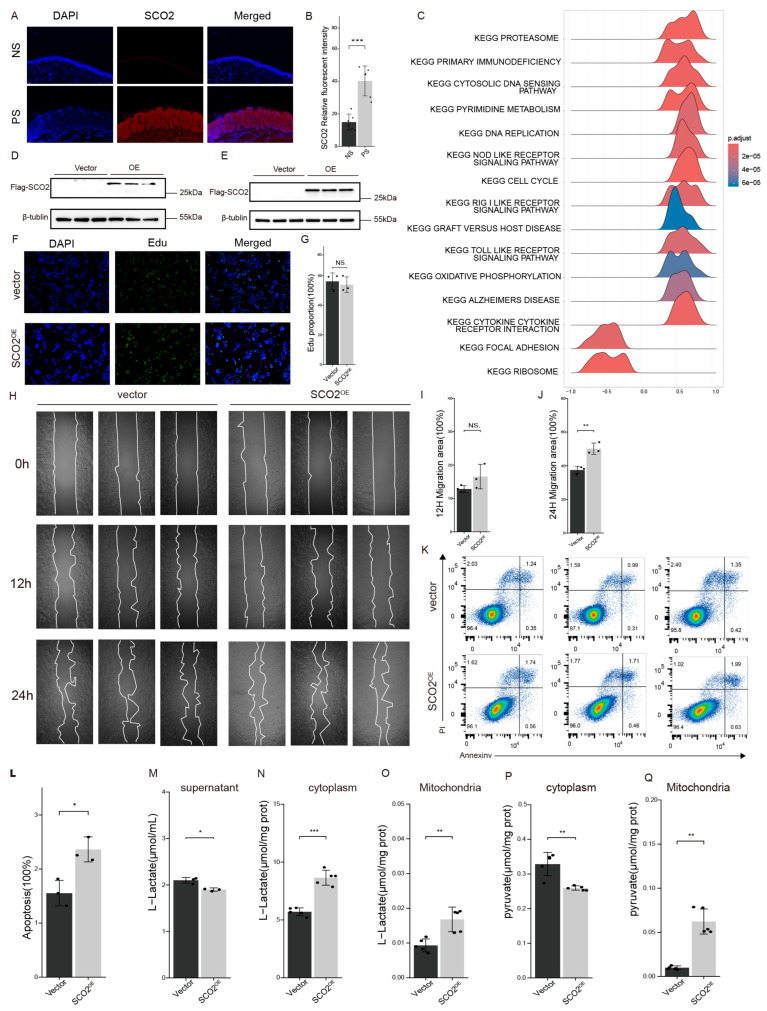
SCO2 is upregulated in psoriasis and regulates keratinocyte migration, apoptosis, and metabolic reprogramming. (**A**,**B**) Validation in tissue samples: (**A**) Representative immunofluorescence images of SCO2 (red) in normal skin and psoriatic lesions. Nuclei were counterstained with DAPI (blue). (**B**) Quantification of the relative fluorescence intensity showing significantly elevated SCO2 protein levels in psoriasis (*n* = 6). (**C**) GSEA of KEGG pathways based on a gene list ranked by correlation with SCO2 in the GSE13355 dataset. The color scale of the ridge plot represents the adjusted *p*-value (p.adj), with the color gradient (from red to blue) indicating the level of statistical significance. (**D**,**E**) Overexpression efficiency: Western blot analysis confirming the successful overexpression of Flag-SCO2 in HaCaT cells. β-tubulin served as the loading control. (**F**,**G**) Cell proliferation assessment. (**F**) Representative images of the EdU incorporation assay in HaCaT cells infected with negative control (Vector) or SCO2 overexpression (OE) lentiviruses. EdU-positive proliferating cells are stained green, and nuclei are counterstained with DAPI (blue). (**G**) Quantification of the proportion of EdU-positive cells. (**H**–**J**) Cell migration: (**H**) Representative images of the wound healing assay at 0, 12, and 24 h. The white lines in (H) outline the migration front of HaCaT cells. (**I**,**J**) Quantitative analysis of the migration area revealed that SCO2 overexpression significantly promoted HaCaT cell migration at 24 h compared to the vector control. (**K**,**L**) Cell apoptosis: (**K**) Flow cytometry analysis using Annexin V-FITC/PI staining. (**L**) The bar graph shows a statistically significant increase in the apoptotic rate of SCO2-overexpressing cells. (**M**–**Q)** Quantification of L-Lactate and Pyruvate levels in different cellular fractions: (**M**) L-Lactate levels in the culture supernatant, (**N**) cytoplasmic L-Lactate, (**O**) mitochondrial L-Lactate, (**P**) cytoplasmic pyruvate, and (**Q**) mitochondrial pyruvate. Data are presented as mean ± SD. Statistical significance was determined by Student’s *t*-test * *p* < 0.05, ** *p* < 0.01, *** *p* < 0.001; NS, not significant.

To investigate the role of SCO2 in psoriasis, we first examined its expression profile. Immunofluorescence analysis confirmed that SCO2 protein levels were significantly elevated in psoriatic lesions, consistent with the upregulated mRNA levels observed in the GSE13355 and GSE14905 datasets (Supplementary Figure S2A,B; Figure 4A,B). To identify biological pathways associated with SCO2 expression, we performed GSEA on gene lists ranked by their correlation coefficients with SCO2 in both the GSE13355 (Figure 4C) and GSE14905 (Supplementary Figure S2C) datasets. SCO2 expression was positively correlated with metabolic pathways, including oxidative phosphorylation pathway. To elucidate the biological function of SCO2, we overexpressed it in HaCaT cells, and Western blot analysis confirmed a significant increase in SCO2 protein levels (Figure 4D,E). SCO2 overexpression did not significantly alter EdU incorporation in these cells (Figure 4F,G). In a subsequent wound healing assay, SCO2 overexpression showed no significant effect on cell migration at 12 h but significantly enhanced migration efficiency by 24 h (Figure 4H–J). Furthermore, flow cytometry analysis using the gating strategy in Supplementary Figure S2D revealed a slight increase in the percentage of apoptotic cells in SCO2-overexpressing cells compared to the control group (Figure 4K,L).

The metabolic impact of SCO2 was assessed by measuring L-lactate and pyruvate levels in subcellular fractions of HaCaT cells. SCO2 overexpression markedly reduced L-lactate secretion into the culture supernatant (Figure 4M). Conversely, L-lactate accumulated in both the cytoplasmic (Figure 4N) and mitochondrial (Figure 4O) compartments. For pyruvate, SCO2 overexpression lowered its cytoplasmic level (Figure 4P) while substantially elevating its mitochondrial content (Figure 4Q). These findings indicate that SCO2 facilitates mitochondrial pyruvate influx and leads to intracellular lactate accumulation, presumably to support oxidative phosphorylation.

### 2.5. Single-Cell Analysis Reveals SCO2-Mediated Activation of the MIF Pathway in Keratinocyte

**Figure 5:**
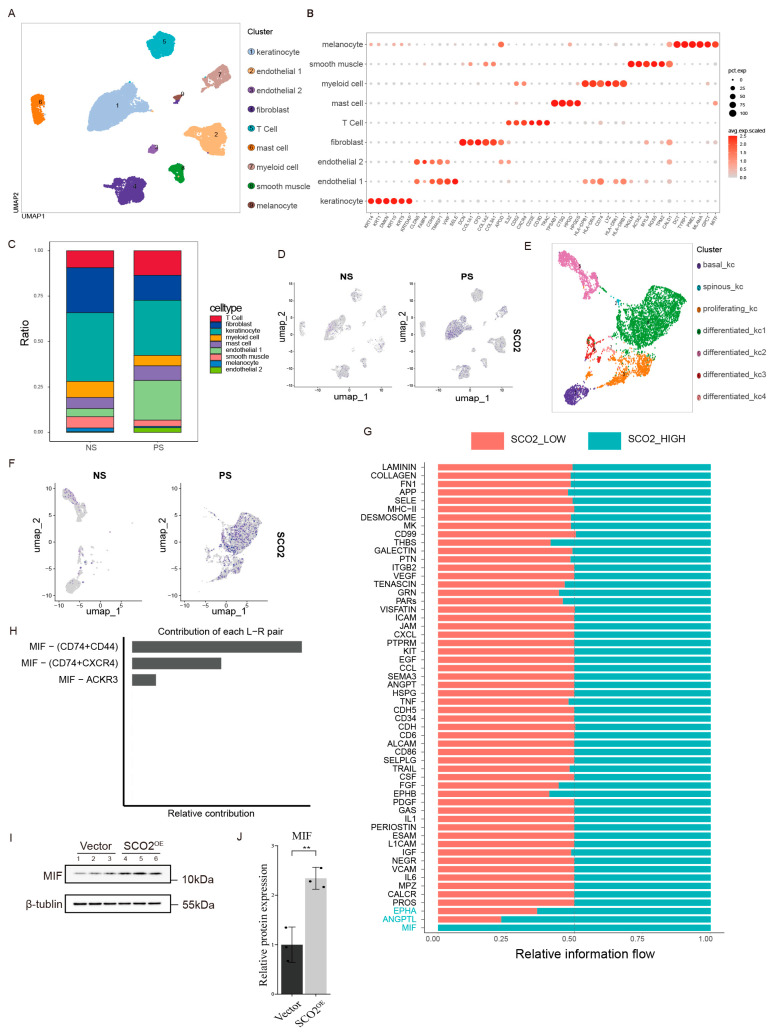
Single-cell transcriptomic analysis identifies the SCO2-MIF signaling axis in psoriatic keratinocytes. (**A**,**B**) UMAP visualization of major cell clusters (**A**) and dot plot of canonical marker genes used for annotation (**B**), the size of each dot represents the percentage of cells expressing the marker gene, while the color intensity indicates the scaled average expression level. (**C**) Bar plot showing the cellular composition proportion in Normal (NS) and Psoriasis (PS) samples. (**D**) Feature plots displaying SCO2 expression distribution in NS and PS groups. (**E**,**F**) Sub-clustering of keratinocytes by UMAP (**E**) and feature plots of SCO2 expression (**F**), (**G**) CellChat analysis showing the relative information flow of signaling pathways. The MIF signaling pathway is predominantly enriched in the SCO2-High group compared to the SCO2-Low group. (**H**) Relative contribution of ligand-receptor (L-R) pairs to the MIF signaling pathway. (**I**,**J**) Western blot analysis (**I**) and quantification (**J**) of MIF protein levels in HaCaT cells infected with vector or SCO2 overexpression (OE) lentiviruses. Data are presented as mean ± SD. Statistical significance was determined by Student’s *t*-test. ** *p* < 0.01.

scRNA-seq data from the GSE150672 dataset, containing 3 NS and 8 PS samples, were analyzed to investigate the critical role of SCO2 in psoriasis pathogenesis. After standard quality control and normalization with the Seurat pipeline, we identified nine distinct cell clusters (Figure 5A). These clusters were annotated as keratinocytes, endothelial cells (subtypes 1 and 2), T cells, fibroblasts, mast cells, myeloid cells, smooth muscle cells, and melanocytes based on canonical marker gene expression (Figure 5B). A comparison of cellular composition revealed an expanded proportion of T cells in the PS group relative to the NS group (Figure 5C), indicating increased T cell infiltration in the disease state. Feature plots demonstrated that SCO2 expression was predominantly localized to keratinocytes (Figure 5D).

We re-clustered the keratinocytes into distinct subpopulations to further characterize the distribution of SCO2 (Figure 5E). The identity of these clusters was confirmed by established markers, including KRT5 and KRT14 for basal keratinocytes, KRT1 and KRT10 for spinous keratinocytes, and MKI67 for proliferating cells (Supplementary Figure S3A). SCO2 expression was highest in the differentiated_kc1 subpopulation, particularly within the PS group (Figure 5F). Using the expression density curve, we defined a cutoff value, indicated by a red dashed line, to stratify cells into SCO2-High and SCO2-Low groups (Supplementary Figure S3B). Subsequently, we compared signaling networks between these groups using CellChat analysis to investigate consequent changes in intercellular communication. This revealed distinct signaling patterns, with the relative information flow analysis showing a marked enrichment and predominance of the MIF signaling pathway in the SCO2-High group (Figure 5G). To pinpoint the specific molecular interactions driving this pathway, we analyzed the relative contribution of ligand-receptor pairs. As shown in Figure 5H, the signaling was primarily mediated by the MIF-(CD74 + CD44) and MIF-(CD74 + CXCR4) complexes. We validated this finding by Western blot, which demonstrated that SCO2 overexpression in HaCaT cells significantly increased MIF protein levels (Figure 5I,J). These results suggest that elevated SCO2 may promote keratinocyte-immune cell interactions via the MIF-mediated crosstalk.

### 2.6. SCO2-High Keratinocytes Promote the Accumulation of CCR7^+^ DCs to Drive T Cell Activation via the MIF Signaling Pathway

**Figure 6:**
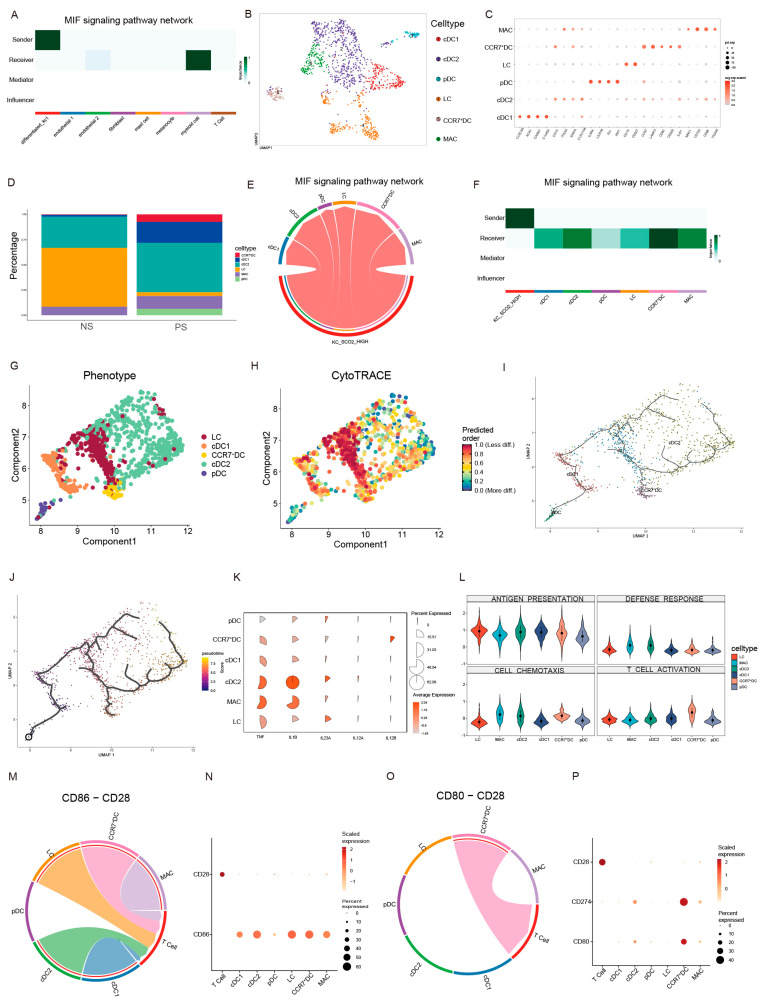
SCO2-High keratinocytes recruit CCR7^+^ DCs via MIF signaling pathway and promote T cell activation. (**A**) Heatmap displaying the signaling roles (Sender, Receiver, Mediator, Influencer) of major cell types in the MIF signaling network. (**B**) UMAP visualization of re-clustered myeloid cell subpopulations. (**C**) Dot plot showing the expression of canonical marker genes used for annotating myeloid subsets. (**D**) Bar plot illustrating the cellular proportion of myeloid subsets in Normal (NS) and Psoriasis (PS) groups. (**E**,**F**) Chord diagram (**E**) and heatmap (**F**) visualizing the inferred MIF signaling interactions between SCO2-High keratinocytes and myeloid subtypes. (**G**–**J**) Trajectory inference analysis. (**G**) Monocle 3 trajectory plot colored by cell type. (**H**) CytoTRACE visualization of predicted differentiation potential. (**I**,**J**) Pseudotime ordering of dendritic cell subsets inferred by Monocle 3. (**K**) Dot plot showing the expression levels of inflammatory cytokines (TNF, IL1B, IL23A, IL12A, IL12B) across myeloid subsets. (**L**) Violin plots of functional scores for antigen presentation, defense response, cell chemotaxis, and T cell activation calculated by AddModuleScore. (**M**–**P**) Visualization of ligand-receptor interactions. Chord diagrams and dot plots displaying the communication probabilities of CD86-CD28 (**M**,**N**) and CD80-CD28 (**O**,**P**) pairs between myeloid subsets and T cells.

To elucidate downstream targets of SCO2 signaling, we analyzed intercellular communication networks. Differentiated_kc1 cells emerged as the primary source of MIF signals targeting the myeloid population (Figure 6A). Sub-clustering of myeloid cells revealed a significant expansion of CCR7^+^ dendritic cells (CCR7^+^ DCs) in psoriasis (Figure 6B–D). CellChat analysis confirmed that SCO2-High keratinocytes interacted most strongly with CCR7^+^ DCs via the MIF pathway (Figure 6E,F).

To determine the developmental origin of these CCR7^+^ DCs, we performed trajectory inference. CytoTRACE analysis indicated that Langerhans cells (LCs) exhibited a lower differentiation state with higher predicted potency, whereas CCR7^+^ DCs represented a more mature state (Figure 6G,H). Pseudotime analysis using Monocle3 suggested a potential developmental trajectory from LCs and plasmacytoid dendritic cells (pDCs) toward the CCR7^+^ DC state (Figure 6I,J).

We further characterized the functional phenotype of CCR7^+^ DCs. Gene expression analysis revealed that CCR7^+^ DCs specifically co-expressed IL12B and IL23A, encoding the intact, secreted cytokine IL-23 (Figure 6K), identifying them as potent producers of this key Th17 response driver. Functional scoring with AddModuleScore confirmed that CCR7^+^ DCs exhibited the highest score for “T cell activation” among all myeloid subsets (Figure 6L). Analysis of ligand-receptor interactions validated this mechanism, showing strong enrichment for the co-stimulatory pairs CD86-CD28 and CD80-CD28 between CCR7^+^ DCs and T cells (Figure 6M–P). These findings indicate that CCR7^+^ DCs are the dominant myeloid subset responsible for priming T cells within the disease microenvironment.

## 3. Discussion

The pathogenesis of psoriasis is fundamentally driven by the intricate crosstalk between hyperproliferative keratinocytes and a complex network of infiltrating immune cells [2,19]. Although metabolic reprogramming is a hallmark of psoriatic keratinocytes, the molecular connections between mitochondrial metabolism and immune crosstalk remain poorly understood [20,21]. Here, we identify SCO2 as a critical regulator linking metabolic changes to immune activation in psoriasis. We demonstrate that SCO2 is upregulated in psoriatic lesions, promoting keratinocyte migration and inducing apoptosis. Mechanistically, SCO2 modulation alters the intracellular distribution of lactate and pyruvate. scRNA-seq analysis further reveals a novel intercellular communication: SCO2-high keratinocytes recruit and activate CCR7^+^ DCs via the MIF signaling pathway, driving T cell activation through IL-23 secretion and co-stimulation.

In vitro assays revealed that SCO2 overexpression significantly enhanced keratinocyte migration—a hallmark of psoriatic plaque expansion—with negligible effects on proliferation. Metabolically, elevated SCO2 promoted mitochondrial pyruvate entry, likely via COX complex modulation, thereby perturbing lactate homeostasis. This resulted in increased intracellular lactate from both mitochondrial and cytosolic sources and decreased extracellular lactate, suggesting impaired lactate efflux or enhanced metabolic consumption. This observation aligns inversely with findings in breast cancer, where the suppression of SCO2 by the HBXIP/miR-183/182 axis resulted in elevated lactate levels in the culture medium [22]. Importantly, our study extends these findings by dissecting the subcellular compartmentalization of lactate, moving beyond the sole assessment of extracellular levels to reveal a specific pattern of intracellular accumulation. These findings indicate that SCO2-overexpressing cells sustain high metabolic flux through concurrent mitochondrial respiration and glycolysis. This metabolic flexibility, driven by SCO2-mediated oxidative phosphorylation, mirrors psoriasis-associated reprogramming and provides the necessary ATP to fuel energy-intensive processes such as migration and inflammatory cytokine secretion. Beyond energy production, SCO2 has been reported to restore the metabolic phenotype in p53-deficient cells, thereby conferring resistance to hypoxia and promoting cell survival [23]. Given the intense metabolic competition inherent to the psoriatic microenvironment, we postulate that SCO2 may also serve as a protective factor, shielding keratinocytes from cell death triggered by local hypoxia or nutrient deprivation. Consistent with this, HIF-1α activation in psoriatic tissue regulates metabolism and coordinates Th17-mediated inflammation [24], and SCO2 is transcriptionally controlled by HIF-1α [25], suggesting SCO2 likely functions as a key metabolic effector in the disease. Benefiting from the advancement of scRNA-seq technology, the resolution for investigating cellular heterogeneity has been significantly improved [26]. Consequently, our single-cell analysis noted elevated SCO2 expression in endothelial cells within psoriatic lesions. This is particularly relevant given the strong association between psoriasis and cardiovascular comorbidities. In the context of diabetic cardiomyopathy, SCO2 overexpression has been shown to accelerate lipid uptake and mitochondrial ROS generation in myocytes, whereas its knockdown attenuates fatty acid metabolism [27]. It is therefore plausible that elevated SCO2 in psoriatic endothelial cells may similarly drive lipid accumulation and oxidative stress, contributing to the vascular dysfunction and cardiovascular risk associated with the disease.

Moreover, prior studies indicate that SCO2 is upregulated under cellular stress, leading to ROS generation, which can trigger cell death if the stress becomes irreversible [28]. Specifically, Madan et al. demonstrated that SCO2 amplifies ROS production and induces apoptosis via the ROS-ASK-1 kinase pathway [17,27]. This mechanism aligns with our observation of elevated apoptosis in SCO2-overexpressing keratinocytes. From these results, we propose that oxidative stress may provoke mitochondrial DNA (mtDNA) leakage into the cytosol. Cytosolic mtDNA could then act as a danger signal, activating the cGAS-STING pathway to exacerbate Th17-type inflammation and drive disease progression [29]. However, we did not directly quantify ROS levels or detect cytosolic mtDNA leakage in this study. These limitations imply that while our data provides evidence linking SCO2 to immunometabolic regulation, the precise downstream mechanisms warrant cautious interpretation and merit further in-depth investigation. Consequently, Future studies should clarify the role of the cGAS-STING axis in the context of SCO2 overexpression in psoriasis.

MIF is a pleiotropic pro-inflammatory cytokine expressed widely in tissues such as the basal epidermis and keratinocytes. In skin physiology, MIF promotes keratinocyte and fibroblast migration, while in pathology it facilitates leukocyte recruitment and amplifies inflammatory responses [30]. Cellular release of MIF occurs rapidly in response to stimuli including LPS, TNF-α, and glucose [31]. Clinically, MIF polymorphisms are significantly linked to psoriasis susceptibility [32,33], and its expression is elevated in inflammatory skin diseases [34,35]. Correspondingly, MIF deficiency markedly ameliorates imiquimod-induced psoriasis-like dermatitis in mice, reducing keratinocyte hyperplasia, inflammatory infiltration, and angiogenesis [36]. Here, we identify a key mechanism whereby SCO2-high keratinocytes drive the recruitment of CCR7^+^ DCs specifically via the MIF-(CD74 + CD44) ligand-receptor interaction. Using single-cell RNA sequencing and CellChat analysis, we determined that SCO2-high keratinocytes are the primary epidermal source of MIF. Our findings indicate that SCO2-mediated metabolic stress or reprogramming in keratinocytes may induce MIF release, thereby promoting leukocyte recruitment and exacerbating inflammation. Furthermore, as illustrated in Figure 6F, macrophages and cDC2s were also identified as recipients of this SCO2-high keratinocyte-derived MIF signaling. These cells concurrently displayed high expression of TNF and IL1B (Figure 6K), suggesting they may act synergistically to sustain the inflammatory microenvironment.

We identified a specific accumulation of CCR7^+^ DCs within psoriatic lesions. This population, referred to in recent single-cell studies as DC.2, LAMP3^+^ DCs, or mregDCs, is defined by co-expression of LAMP3, CCR7, and multiple chemokines, as well as high levels of IL32 [37,38,39,40]. Functionally, these CCR7^+^ DCs are highly pathogenic, acting as a major of bioactive IL-23 (IL12B + IL23A) (Figure 6K) and expressing elevated levels of the co-stimulatory molecules CD80 and CD86 [41] (Figure 6N,P). Critically, the persistence of CCR7^+^ DCs is associated with disease relapse and treatment resistance. Sun et al. recently showed that in psoriasis patients treated with glucocorticoids, CCR7^+^ DCs were significantly reduced but rebounded upon relapse, whereas they remained stable in non-responders, correlating with therapeutic resistance [41,42]. Similarly, in atopic dermatitis (AD), LAMP3^+^ DCs can persist for up to a year after clinical resolution [39]. Together, these findings establish CCR7^+^ DCs as a central component of disease chronicity and recurrence. Concerning their developmental origin, a recent study demonstrated that pDCs can be reprogrammed toward a cDC-like phenotype featuring upregulated LAMP3 and CCR7 following inflammatory stimulation [43]. In line with this, our trajectory analysis positioned pDCs and Langerhans cells in a less differentiated state relative to CCR7^+^ DCs, implying a potential developmental transition whereby stimulated pDCs or LCs mature into the CCR7^+^ DC phenotype, since IL-23 is the master regulator of the Th17 axis, and CD80/86 delivers the essential second signal for T cell priming.

Despite the significant insights provided by this study, several limitations should be acknowledged and addressed in future investigations. While we identified a retention of lactate and pyruvate, we did not perform real-time metabolic flux analysis. To deeply elucidate the mechanism of intracellular lactate accumulation—whether it stems from enhanced glycolytic demand or impaired efflux—future work should employ Seahorse XF analysis to measure the Oxygen Consumption Rate (OCR) and Extracellular Acidification Rate (ECAR). Furthermore, tracing lactate transport and utilization efficiency is critical to clarify the precise dynamics of this ‘lactate retention’ state. Beyond metabolic characterization, further investigation is required to understanding the specific molecular mechanisms by which this intracellular lactate accumulation triggers MIF secretion. Finally, the lack of in vivo validation restricts our understand of the systemic effects. To substantiate our findings, future studies should utilize keratinocyte-specific SCO2 conditional knockout mice in an imiquimod (IMQ)-induced psoriasis model. This will allow us to directly assess whether SCO2 deletion alleviates psoriatic phenotypes and specifically reduces the infiltration of CCR7^+^ DCs in the epidermal microenvironment.

## 4. Materials and Methods

### 4.1. Identification of Differentially Expressed Genes (DEGs) and Functional Enrichment Analysis

Transcriptomic datasets from the skin of psoriasis patients were acquired from the Gene Expression Omnibus (GEO) database (https://www.ncbi.nlm.nih.gov/geo/, accessed on 14 February 2025). This study utilized two datasets, GSE13355 [44] and GSE14905 [45]. The subsequent analysis incorporated a total of 91 psoriatic skin (PS) samples and 85 normal skin (NS) controls, which consisted of 58 PS and 64 NS samples from GSE13355, and 33 PS and 21 NS samples from GSE14905 (Supplementary Table S1).

Transcriptomic data processing and differential expression analysis were performed independently for each dataset. Raw data were pre-processed using the RMA (Robust Multi-array Average) algorithm via the affy R package (version 1.84.0). Subsequently, data normalization was conducted using the normalizeBetweenArrays function from the limma R package (version 3.62.2) to ensure comparability. The design matrix was constructed as~Group (Psoriasis sink vs. Normal skin). Differential expression analysis was then conducted using the limma R package (version 3.62.2) [46]. DEGs were defined by an adjusted *p*-value < 0.05 and an absolute log2 fold change (|log2FC|) > 0.5. The distribution of these DEGs was visualized with the “ggplot2” package (version 3.5.1).

Functional differences between psoriasis patients and healthy controls were identified through Kyoto Encyclopedia of Genes and Genomes (KEGG) pathway enrichment analysis and Gene Set Enrichment Analysis (GSEA) using the “clusterProfiler” package (version 4.14.4) [47]. Gene annotation was performed with the “org.Hs.eg.db” package (version 3.20.0). The GSEA utilized a custom gene set related to lactate metabolism (Supplementary Table S2). For the KEGG analysis, statistical significance was defined as an adjusted *p*-value of less than 0.05, and results were visualized using the “ggplot2” package (version 3.5.1). In the GSEA, a Normalized Enrichment Score (NES) > 1 and a False Discovery Rate (FDR; represented by the Benjamini–Hochberg adjusted *p*-value) < 0.25 were considered statistically significant, and enrichment plots were generated using the “enrichplot” package (version 1.26.6).

### 4.2. Weighted Gene Co-Expression Network Analysis (WGCNA) and Identification of Lactate-Related Hub Genes

Weighted Gene Co-expression Network Analysis (WGCNA) was conducted with the “WGCNA” R package (version 1.73) to identify biologically relevant gene modules and investigate their association with the disease phenotype [48]. We selected the top 10,000 genes exhibiting the highest variance for the analysis. An optimal soft-thresholding power (β) was determined using the ‘pickSoftThreshold’ function to achieve a scale-free network topology, targeting a scale-free topology fit index (R2) of 0.9. The adjacency matrix was then converted into a Topological Overlap Matrix (TOM), from which a dissimilarity measure (1-TOM) was derived. Co-expression modules were identified through hierarchical clustering and dynamic tree cutting, and modules with similar expression profiles were subsequently merged. For further investigation, we focused on genes within the module demonstrating the strongest correlation with psoriasis.

A total of 353 lactate-related genes (LRGs) were obtained from the Molecular Signatures Database (MSigDB) using the search term “lactate” (https://www.gsea-msigdb.org/gsea/msigdb/; accessed 30 November 2024) [49] (Supplementary Table S2). To identify candidate genes, we intersected the differentially expressed genes (DEGs) from the GSE13355 and GSE14905 datasets, the genes from the most significant WGCNA module, and the identified LRGs using the “VennDiagram” R package (version 1.7.3).

To identify lactate-related hub genes, we applied three machine learning algorithms: Least Absolute Shrinkage and Selection Operator (LASSO) regression with the “glmnet” package (version 4.1-8) [50], Support Vector Machine-Recursive Feature Elimination (SVM-RFE) using the “e1071” (version 1.7-16), “caret” (version 7.0-1), and “kernlab” packages (version 0.9-33) [51], and Random Forest (RF) with the “randomForest” package (version 4.7-1.2) [52]. Each algorithm was run independently on both the GSE13355 and GSE14905 datasets. We then used Venn analysis to intersect the candidate genes selected by each method across both datasets, thereby defining a final set of core lactate-related hub genes.

### 4.3. Single-Cell RNA Sequencing (scRNA-Seq) Data Analysis

The scRNA-seq dataset GSE150672 [53], comprising skin samples from eight patients with psoriatic lesions and three normal controls, was obtained from the GEO database (Supplementary Table S1). Data processing and analysis were performed with the Seurat R package (version 5.2.1) [54]. Quality control involved filtering out low-quality cells, specifically those with fewer than 300 detected genes or a mitochondrial gene expression percentage exceeding 10%. The filtered gene expression matrix was normalized using the “LogNormalize” function with default parameters. Highly variable features were then identified with the “FindVariableFeatures” function. Following this, the data were scaled using the “ScaleData” function. Dimensionality reduction was carried out via Principal Component Analysis (PCA) using the “RunPCA” function. Unsupervised clustering, based on the top principal components, was performed with the “FindNeighbors” and “FindClusters” functions. Finally, cell clusters were visualized in two dimensions using the “RunUMAP” function.

### 4.4. Module Score Calculation and Visualization

Gene expression module scores for individual cells were calculated with the “AddModuleScore” function in Seurat. The distribution of these scores was then assessed and visualized using violin plots created by the ggviolin function from the “ggpubr” package (version 0.6.0).

### 4.5. Cell Type Annotation

Cell types were annotated according to canonical marker gene expression. T cells were identified using IL32, TRAC, CXCR4, CD3D, CD3E, and CD52; melanocytes by DCT, TYRP1, PMEL, MLANA, QPCT, and MITF; myeloid cells by LYZ, HLA-DPB1, HLA-DRA, CD74, HLA-DPA1, and HLA-DRB1; keratinocytes by KRT5, KRT14, DMKM, KRT1, KRT10, and KRTDAP; fibroblasts by DCN, COL3A1, COL1A2, COL1A1, CFD, and APOD; endothelial cells by CLDN5, FABP4, CDH5, TM4SF1, VWF, and SELE; mast cells by TPSAB1, CTSG, HPGD, and HPGDS; and smooth muscle cells by TAGLN, ACTA2, MYL9, RGS5, TPM2, and CALD1. Keratinocytes and myeloid cells were subsequently sub-clustered and annotated based on the expression of canonical lineage markers. For keratinocytes, sub-clusters were identified as basal (KRT5, KRT14), spinous (KRT1, KRT10), and proliferating (MKI67, TOP2A) cells. Notably, the differentiated subpopulations were characterized by the distinct expression patterns of various KRT genes and S100A7/S100A8/S100A9. For the myeloid lineage, cells were annotated as cDC1 by CLEC9A, XCR1, CADM1 and C1orf54; cDC2 by CD1C, ITGAX, SIRPA and CLEC10A; pDC by IL3RA, IRF7, LILRA4 and IGJ; Langerhans cells(LC) by CD1A and CD207; CCR7^+^ DCs by CCR7, LAMP3, CD80 and CD200, IL4I1; and macrophages(MAC) by CD68, CD163, MRC1 and ITGAM.

### 4.6. Cell Maturation and Trajectory Analysis

To predict the differentiation potential of dendritic cell (DC) subpopulations, we employed the CytoTRACE R package (version 0.3.3). Pseudotime analysis was subsequently conducted with Monocle3 (version 1.3.7) to delineate DC maturation dynamics [55]. A CellDataSet (CDS) object was first created using the “new_cell_data_set” function. The data were preprocessed and normalized through log-transformation with the “preprocess_cds” function. Following dimensionality reduction via PCA, cells were clustered using the Leiden algorithm. The trajectory graph was then learned, and the “plasmacytoid dendritic cell” (pDC) cluster was manually designated as the root node with the “order_cells” function to infer pseudotime ordering.

### 4.7. Cell–Cell Communication Analysis

Intercellular communication networks were inferred, analyzed, and visualized with the CellChat R package (version 2.1.2) [56]. The gene expression data for the identified cell clusters were used to construct a CellChat object. For ligand-receptor interaction analysis, we employed the CellChatDB.human database as a reference of known ligand-receptor pairs. Communication probabilities were computed, and significant interactions were identified via a permutation test. The aggregated cell–cell communication networks were then visualized to depict the number of interactions and their strength between different cell types.

### 4.8. Cell Culture and Generation of Stable Overexpression Cell Lines

HaCaT cells are a spontaneously immortalized human keratinocyte line widely accepted as an in vitro model for studying psoriasis pathogenesis due to their preservation of key functional characteristics similar to normal keratinocytes [57]. These cells were obtained from Cyagen Biosciences (Suzhou, China; Agreement No. STSKO220323CBC1) and cultured in DMEM (Gibco; Thermo Fisher Scientific, Inc., Waltham, MA, USA; Cat# C11995500BT) supplemented with 10% fetal bovine serum (FBS; Gibco; Thermo Fisher Scientific, Inc., Waltham, MA, USA) and 1% penicillin-streptomycin. They were maintained in a humidified incubator at 37 °C with 5% CO_2_.

Lentiviruses for SCO2 overexpression and the corresponding negative control vectors were obtained from Genechem Co., Ltd. (Shanghai, China). To generate stable cell lines, HaCaT cells were plated in 6-well plates and allowed to reach 50–60% confluence. These cells were then infected with either the recombinant lentivirus or the control virus using RNAi-Mate transfection reagent (GenePharma, Shanghai, China) as per the manufacturer’s instructions. After 48 h, the medium was refreshed with complete medium. Selection of stably infected cells proceeded by replacing the culture medium every three days with medium containing 2 µg/mL puromycin (Biosharp, Hefei, China; Cat# BL528A). The resulting stable cell populations were collected for subsequent analyses, which comprised apoptosis assays, EdU proliferation assays, wound healing assays, lactate/pyruvate measurements, and protein expression analysis.

### 4.9. EdU Cell Proliferation Assay

Cell proliferation was assessed with the EdU Cell Proliferation Imaging Kit (Abbkine Scientific Co., Ltd., Wuhan, China; Cat# KTA2030) according to the manufacturer’s protocol. Cells were seeded onto sterile coverslips and allowed to adhere overnight. They were then incubated for 2 h at 37 °C in culture medium containing 10 µM EdU. Following fixation and permeabilization, a Click-iT reaction mixture was applied for 30 min at room temperature in the dark. After three washes with PBS, nuclei were counterstained with DAPI (KeyGEN BioTECH, Cat# KGA1529-25). The coverslips were mounted on glass slides, and images were captured using a Pannoramic MIDI II digital scanner (3DHISTECH, Budapest, Hungary). The percentage of EdU-positive cells was quantified with ImageJ software (version 1.54p).

### 4.10. Wound Healing Assay

Cells were seeded in 6-well plates and cultured until they reached approximately 80–90% confluence. A sterile 200 μL pipette tip was then used to create a linear scratch across the cell monolayer. After gently washing with PBS to remove debris, the cells were incubated in serum-free medium. The wound area was imaged at 0, 12, and 24 h using an inverted microscope. ImageJ software (version 1.54p) was used to quantify the wound closure area, and the migration rate was calculated as the percentage of wound closure.

### 4.11. Apoptosis Assay

Apoptosis was assessed with the FITC Annexin V Apoptosis Detection Kit I (BD Pharmingen, San Diego, CA, USA; Cat# 556547) following the manufacturer’s protocol. After 24 h in culture, cells were harvested, washed twice with cold PBS, and resuspended in binding buffer. They were then stained with Annexin V-FITC and Propidium Iodide (PI) for 15 min at room temperature in the dark. Samples were analyzed on a CytoFLEX flow cytometer (Beckman Coulter Coulter, Brea, CA, USA) using CytExpert software (version 2.4). The resulting data were analyzed with FlowJo software (version 10.8.1).

### 4.12. Mitochondria and Cytoplasm Extraction

Mitochondria and cytoplasmic fractions were isolated with a Mitochondria Extraction Kit (Solarbio, Beijing, China; Cat# SM0020) following the manufacturer’s protocol. Approximately 5 × 10^7^ cells were harvested, resuspended in 1 mL of Lysis Buffer, and homogenized on ice with 40 strokes in a pre-cooled Dounce homogenizer. The homogenate was centrifuged at 1000× *g* for 5 min at 4 °C to pellet nuclei and cellular debris. After collecting the supernatant, a further centrifugation at 12,000× *g* for 10 min at 4 °C separated the cytosolic and mitochondrial components. The cytosolic fraction (supernatant) was transferred to a new tube. The mitochondrial pellet was washed twice with Wash Buffer and resuspended in Storage Buffer. Both fractions were either analyzed immediately or stored at −80 °C for later use.

### 4.13. Measurement of L-Lactate and Pyruvate Levels

L-lactate and pyruvate levels were measured in the culture supernatant, cytoplasm, and mitochondria with specific assay kits.

For extracellular L-lactate analysis, SCO2-overexpressing and vector control cells were incubated overnight at 37 °C in serum-free MEM. The culture supernatant was subsequently collected for measurement. Intracellular L-lactate was analyzed in mitochondrial and cytosolic fractions, which were isolated using the procedure detailed in the previous section.

L-Lactate levels were determined with the L-Lactate Content Assay Kit (Solarbio, Beijing, China; Cat# BC2235), while pyruvate levels were measured using the Pyruvate Content Assay Kit (Solarbio; Cat# BC2205), in strict accordance with the manufacturer’s protocols. The concentrations of L-lactate and pyruvate in the mitochondrial and cytosolic fractions were then normalized to the total protein concentration of each sample.

### 4.14. Immunofluorescence (IF) Staining

SCO2 expression in skin tissues was evaluated using a Three-Color Four-Channel Multiplex Fluorescence Staining Kit (Aifang Biological, Changsha, China; Cat# AFIHC024) configured for single-marker immunofluorescence detection. Formalin-fixed paraffin-embedded (FFPE) sections of psoriatic and normal skin were deparaffinized, rehydrated, and subjected to antigen retrieval. Endogenous peroxidase activity was quenched with 3% H_2_O_2_, and non-specific binding sites were blocked with 3% BSA to minimize background signal. The sections were subsequently incubated with a primary antibody against SCO2 (1:1000; Proteintech, Wuhan, China; Cat# 21223-1-AP) according to the manufacturer’s protocol. Finally, images were acquired using a Pannoramic MIDI II digital scanner (3DHISTECH, Budapest, Hungary) with fixed exposure parameters to ensure quantitative comparability.

### 4.15. Western Blotting

Total protein was extracted from cells with RIPA Lysis Buffer (Biosharp, Hefei, China; Cat# BL504A) containing PMSF (Beyotime; Shanghai, China; Cat# ST506) and a Protease and Phosphatase Inhibitor Cocktail (Beyotime; Shanghai, China; Cat# P1008). Protein concentrations were quantified, and equal amounts of protein were resolved on 10% SDS-PAGE gels and transferred to PVDF membranes. After blocking with 5% BSA for 1 h at room temperature, the membranes were incubated with specific primary antibodies overnight at 4 °C. The primary antibodies included anti-SCO2 (Proteintech; Wuhan, China; Cat# 21223-1-AP; diluted 1:2000), anti-MIF (Boster; Pleasanton, CA, USA; Cat# PB9274; diluted 1:2000), and a Recombinant Anti-Flag Tag antibody (Servicebio; Wuhan, China; Cat# GB15939-100; diluted 1:5000). Protein bands were visualized using SuperKine™ West Femto Maximum Sensitivity Substrate (Abbkine; Wuhan, China; Cat# BMU102) following incubation with HRP-conjugated secondary antibodies.

### 4.16. Statistical Analysis

Statistical analyses were conducted with R software (version 4.4.2). In vitro experiments were performed with a minimum of three independent replicates. For comparisons between two groups, Student’s *t*-test was applied to parametric data and the Wilcoxon test to non-parametric data. Statistical significance was defined as a *p*-value of less than 0.05.

## 5. Conclusions

In summary, our study reveals that SCO2 is upregulated in psoriatic tissues and functionally modulates cellular metabolism, leading to intracellular lactate accumulation. Importantly, single-cell analysis identifies CCR7^+^ DCs as a critical subset engaged in MIF-mediated crosstalk with SCO2-high keratinocytes. We demonstrate that this subset serves as a major source of bioactive IL-23 and facilitates T cell activation, thereby contributing to the pathogenesis of psoriasis. Our findings, together with future validation studies, suggest that SCO2 represents a promising therapeutic target, offering new insights for optimizing personalized treatment strategies in psoriasis.

## Data Availability

The datasets used and analyzed during the current study are available from Gene Expression Omnibus database. Accession Number: GSE13355, https://www.ncbi.nlm.nih.gov/geo/, accessed on 14 February 2025; Accession Number: GSE14905, https://www.ncbi.nlm.nih.gov/geo/, accessed on 14 February 2025; Accession Number: GSE150672, https://www.ncbi.nlm.nih.gov/geo/, accessed on 14 February 2025.

## Supplementary Materials

The following supporting information can be downloaded at: https://www.mdpi.com/article/10.3390/ijms27031397/s1.
