# Supplementary material for: Bulk and Single-Cell Transcriptomics Reveal That SCO2 Drives Psoriasis via Activating CCR7+ Dendritic Cell
Source: Int J Mol Sci. 2026 Jan 30;27(3):1397. doi: 10.3390/ijms27031397 (PMC12897791; doi:10.3390/ijms27031397)
Supplement: Supplementary file 1 [file ijms-27-01397-s001.zip › Supplementary Figure S1.pdf]

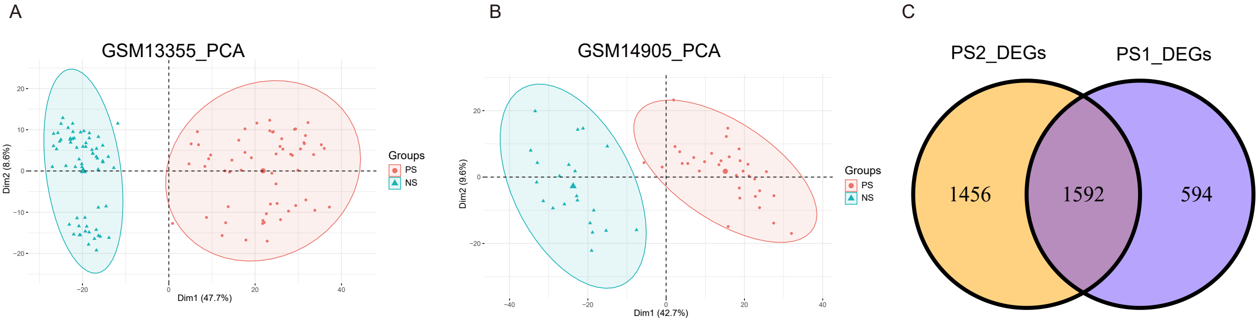

Supplementary Figure S1. Quality control and consistency analysis of transcriptomic datasets.
(A-B) PCA plots visualizing the spatial distribution and clustering of samples in the GSE13355 (A) and GSE14905 (B) datasets. Red circles represent psoriatic lesional skin (PS), and blue triangles represent normal skin (NS). The clear separation indicates distinct gene expression profiles. **(C)** Venn diagram displaying the intersection of differentially expressed genes (DEGs) between GSE14905 (labeled PS2_DEGs) and GSE13355 (labeled PS1_DEGs). The substantial overlap of 1,592 genes demonstrates statistically significant consistency between the two independent cohorts (Hypergeometric test, $P < 2.2 \times 10^{-16}$).
