# Supplementary material for: Bulk and Single-Cell Transcriptomics Reveal That SCO2 Drives Psoriasis via Activating CCR7+ Dendritic Cell
Source: Int J Mol Sci. 2026 Jan 30;27(3):1397. doi: 10.3390/ijms27031397 (PMC12897791; doi:10.3390/ijms27031397)
Supplement: Supplementary file 1 [file ijms-27-01397-s001.zip › Supplementary Figure S2.pdf]

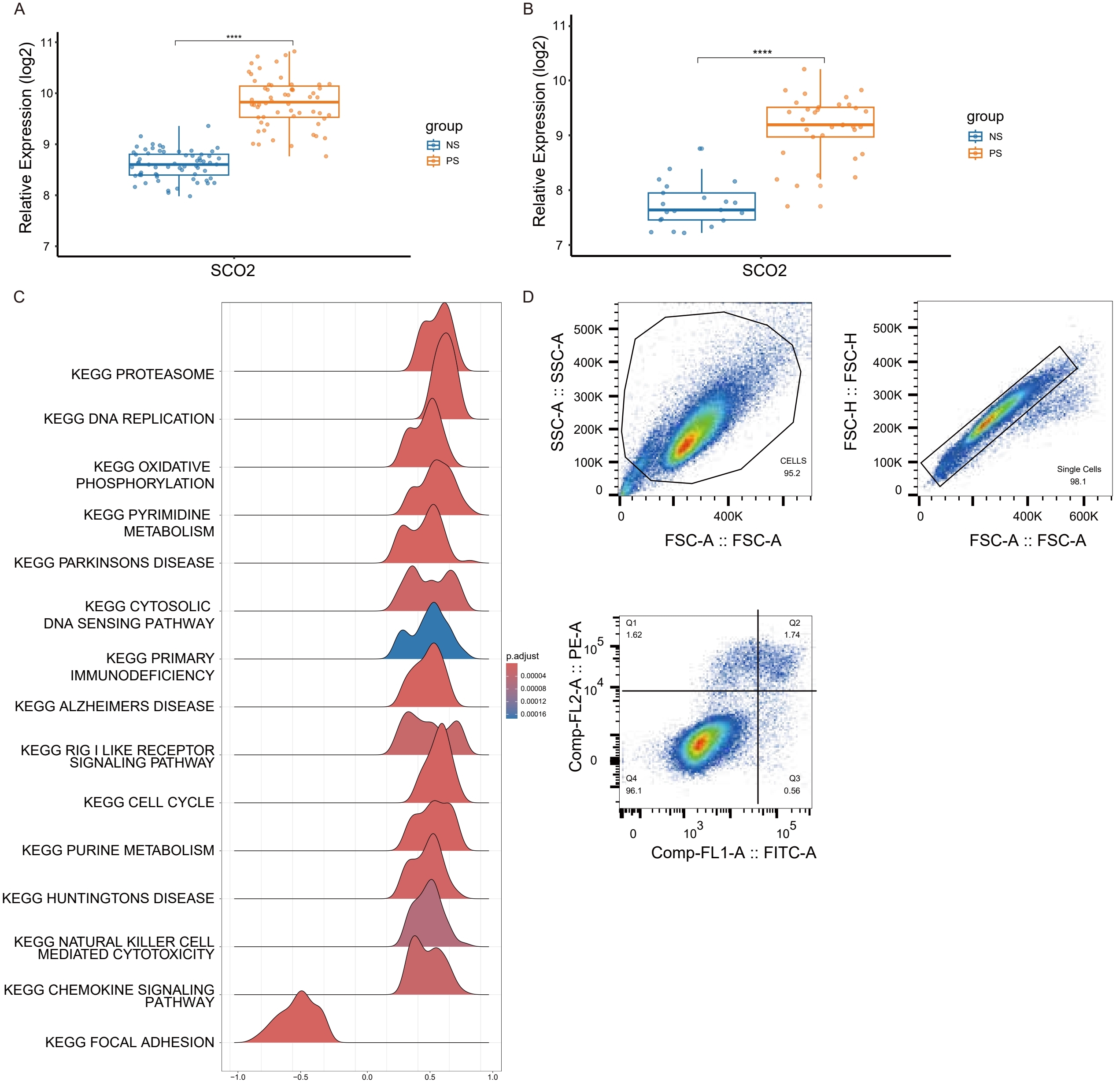

Supplementary Figure S2. Validation of SCO2 expression, pathway enrichment, and flow cytometry gating strategy.
(A-B) Box plots showing the mRNA expression levels of SCO2 in normal skin (NS) and psoriatic lesions (PS) from the GSE13355 (A) and GSE14905 (B) datasets. The Y-axis represents the normalized gene expression intensity on a log2 scale. **(C)** Ridge plot of Gene Set Enrichment Analysis (GSEA) illustrating KEGG pathways significantly enriched in the SCO2-high phenotype in the GSE14905 dataset. **(D)** Representative gating strategy used for flow cytometry analysis.
