# Supplementary material for: Bulk and Single-Cell Transcriptomics Reveal That SCO2 Drives Psoriasis via Activating CCR7+ Dendritic Cell
Source: Int J Mol Sci. 2026 Jan 30;27(3):1397. doi: 10.3390/ijms27031397 (PMC12897791; doi:10.3390/ijms27031397)
Supplement: Supplementary file 1 [file ijms-27-01397-s001.zip › Supplementary Figure S3.pdf]

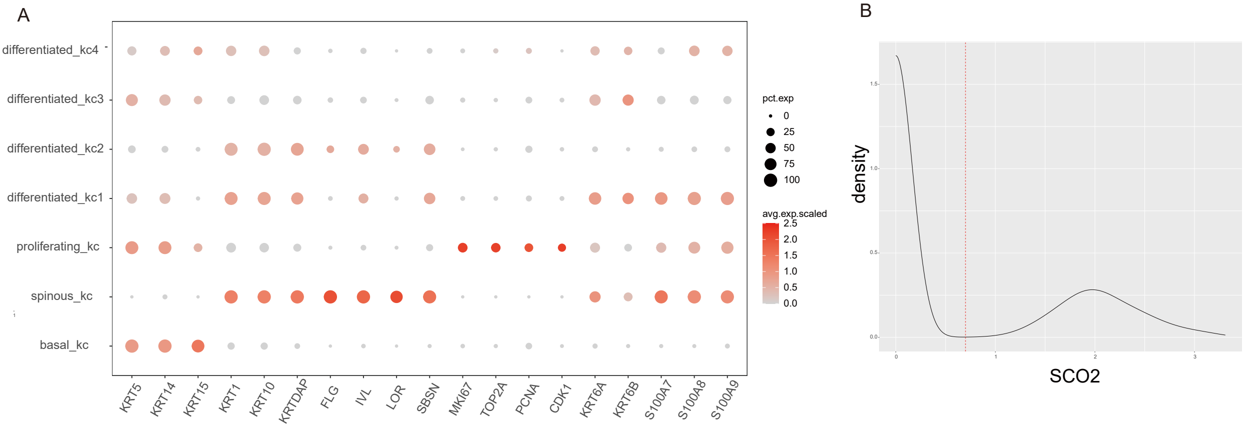

Supplementary Figure S3. Annotation of keratinocyte subpopulations and stratification based on SCO2 expression.
(A) Dot plot visualizing the expression of canonical marker genes used to identify and annotate distinct keratinocyte clusters. **(B)** Density plot showing the distribution of SCO2 expression within the differentiated_kc1 subpopulation. The red dashed line indicates the cutoff value used to stratify cells into SCO2-High and SCO2-Low groups for downstream analysis.
